
JOURNAL INFORMATION
==============================
NLM Title Abbreviation: ChemistryOpen
Journal ID: ChemistryOpen
NLM Journal ID: 101594811
Journal ID: open

ChemistryOpen

EISSN: 2191-1363
Publisher: Wiley

ARTICLE INFORMATION
==============================
PMCID: PMC3646429
PMID: 24551532
DOI: 10.1002/open.201390009
Article version: 1
Subjects: News

Spotlights on our sister journals: ChemistryOpen 2/2013

Print publication date: 2013 Apr
Electronic publication date: 2013 Apr 23
Volume: 2
Issue: 2
First page: 46
Last page: 49
Copyright: Copyright © 2013 WILEY-VCH Verlag GmbH & Co. KGaA, Weinheim
Copyright year: 2013
License: Re-use of this article is permitted in accordance with the Creative Commons Deed, Attribution 2.5, which does not permit commercial exploitation.
License URL: https://creativecommons.org/licenses/by/2.5/

==============================
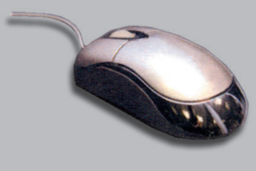

On these pages, we feature a selection of the excellent work that has recently been published in our sister journals. If you are reading these pages on a computer, click on any of the items to read the full article. Otherwise please see the DOIs for easy online access through Wiley Online Library.

### Carbon Dots

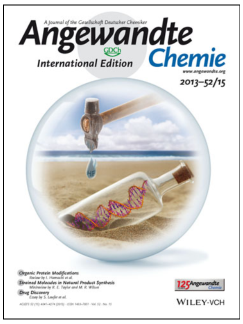

*S. Zhu, Q. Meng, L. Wang, J. Zhang, Y. Song, H. Jin, K. Zhang, H. Sun, H. Wang, B. Yang**

**Highly Photoluminescent Carbon Dots for Multicolor Patterning, Sensors, and Bioimaging**

**Shine on you crazy dots**: A rapid and high-output strategy for the fabrication of polymer-like carbon dots (CDs) with quantum yields as high as ca. 80 % is presented. This value is the highest reported to date for fluorescent carbon-based materials, and gives promise for their application in multicolor-patterning and biosensors.

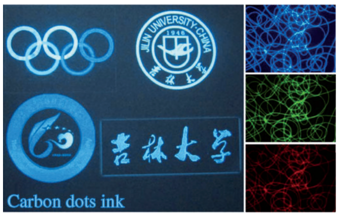

*Angew. Chem. Int. Ed.*
DOI: **10.1002/anie.201300519**

### Anion Recognition

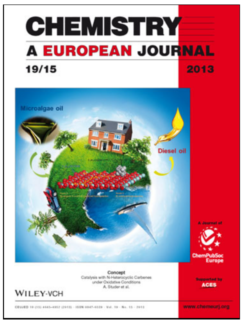

*N. Ousaka, Y. Takeyama, E. Yashima**

**Anion-Driven Reversible Switching of Metal-Centered Stereoisomers in Metallopeptides**

**Anion-triggered chiral switch**: Reversible switching between *fac* and *mer* isomers in tris(2,2′-bipyridine)iron(II) complexes, the ligands of which are substituted at the 5-position with various peptides of different lengths and sequences, has been achieved (see figure). The remote stereocontrol at the $Fe^{II}$ center by a domino-type chiral information transfer along an achiral peptide main-chain was observed even over 3 nm (50 bond lengths).

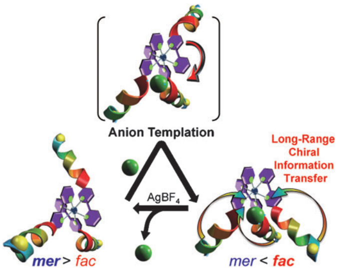

*Chem. Eur. J.*
DOI: **10.1002/chem.201300361**

### C–H Activation

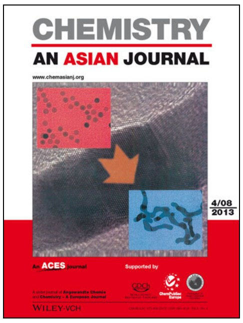

*H. Guo, Z. Chen, F. Mei, D. Zhu, H. Xiong, G. Yin**

**Redox Inactive Metal Ion Promoted C–H Activation of Benzene to Phenol with $Pd^{II}$(bpym): Demonstrating New Strategies in Catalyst Designs**

**Al in**: A new strategy was introduced to modify the electronics and steric hindrance of the $Pd^{II}$ ion in order to change its reactivity towards benzene hydroxylation. In trifluoroacetic acid, free $Pd^{II}$ ions provide dominantly biphenyl, with phenol as minor product. Ligation of bpym to the $Pd^{II}$ ion results in its deactivation with regard to benzene functionalization. The addition of the redox inactive $Al^{III}$ ion to the $Pd^{II}$(bpym) complex recovers its catalytic activity, and alters the reactivity of $Pd^{II}$ ion from benzene coupling to hydroxylation.

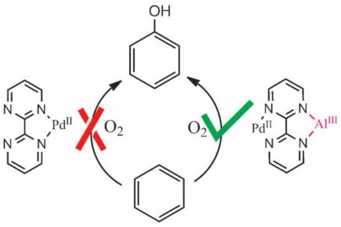

*Chem. Asian J.*
DOI: **10.1002/asia.201300003**

© 2013 Wiley-VCH Verlag GmbH & Co. KGaA, Weinheim

### Selenazolines

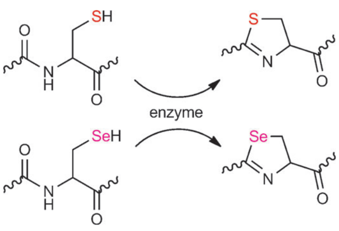

*J. Koehnke, F. Morawitz, A. F. Bent, W. E. Houssen, S. L. Shirran, M. A. Fuszard, I. A. Smellie, C. H. Botting, M. C. M. Smith, M. Jaspars,* J. H. Naismith**

**An Enzymatic Route to Selenazolines**

**Ringing the changes**: Selenazolines have applications in medicinal chemistry, but their synthesis is challenging. We report a new convenient and less toxic route to these heterocycles that starts from commercially available selenocysteine. The new route depends on a heterocyclase enzyme that creates oxazolines and thiazolines from serines/threonines and cysteines.

*ChemBioChem*
DOI: **10.1002/cbic.201300037**

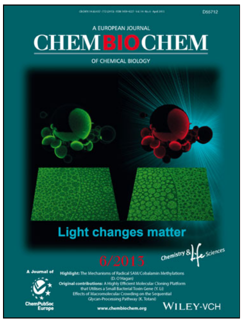

### Molecular Switches

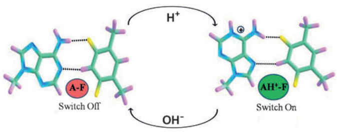

*A. K. Jissy, S. Konar, A. Datta**

**Molecular Switching Behavior in Isosteric DNA Base Pairs**

**Paired up**: The structures and proton-coupled behavior of adenine–thymine and a modified base pair containing a thymine isostere, adenine–difluorotoluene (A-F), are studied in different solvents by dispersion-corrected density functional theory. DNA sequences capable of changing their sequence conformation on protonation are used in the construction of pH-based molecular switches (see picture).

*ChemPhysChem*
DOI: **10.1002/cphc.201201083**

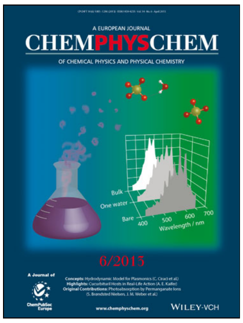

### Drug Discovery

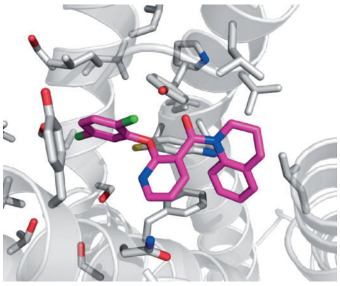

*R. E. Martin,* C. Bissantz, O. Gavelle, C. Kuratli, H. Dehmlow, H. G. F. Richter, U. Obst Sander, S. D. Erickson, K. Kim, S. L. Pietranico-Cole, R. Alvarez-Sánchez, C. Ullmer*

**2-Phenoxy-nicotinamides are Potent Agonists at the Bile Acid Receptor GPBAR1 (TGR5)**

**Potency with potential**: 2-Phenoxynicotinamides were identified as potent agonists at the GPBAR1 receptor, a target in the treatment of obesity, type 2 diabetes and metabolic syndrome. Extensive structure–activity relationship studies supported by homology modeling and docking resulted in the identification of optimized GPBAR1 agonists, potent against both human and mouse receptors, endowed with favorable physicochemical properties and good metabolic stability.

*ChemMedChem*
DOI: **10.1002/cmdc.201200474**

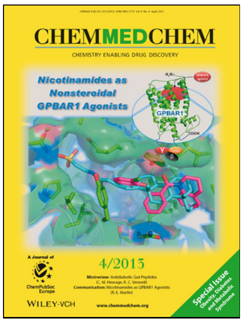

### Renewables

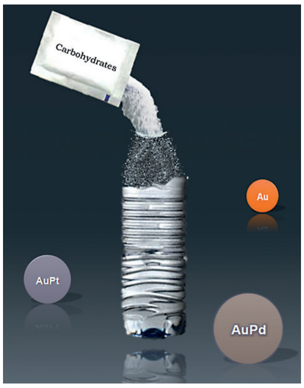

*A. Villa, M. Schiavoni, S. Campisi, G. M. Veith, L. Prati**

**Pd-modified Au on Carbon as an Effective and Durable Catalyst for the Direct Oxidation of HMF to 2,5-Furandicarboxylic Acid**

**Mixed noblility**: We show that the modification of a gold/carbon catalyst with platinum or palladium produces stable and recyclable catalysts for the selective oxidation of 5-hydroxymethylfurfural (HMF) to 2,5-furandicarboxylic acid (FDCA): the support and nanoparticle chemistry directly mediate the selective oxidation of terminal hydroxyl groups in bio-derived HMF. This finding is a significant advance over current conversion technology because of the technological importance of FDCA.

*ChemSusChem*
DOI: **10.1002/cssc.201200778**

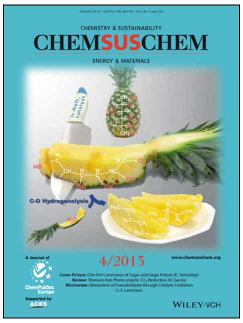

© 2013 Wiley-VCH Verlag GmbH & Co. KGaA, Weinheim

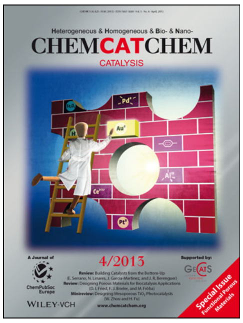

### Mesoporous Catalysts

*S. Das, A. Goswami, N. Murali, T. Asefa**

**Efficient Tertiary Amine/Weak Acid Bifunctional Mesoporous Silica Catalysts for Michael Addition Reactions**

**Dual activity**: We report the synthesis and efficient catalytic properties of acid–base bifunctional mesoporous silica catalyst comprising tertiary amine and optimized concentration of surface silanol (weak acid) groups for the Michael addition reactions between various *trans*-β-nitrostyrenes and active methylene compounds, such as malononitrile, acetylacetone, and diethylmalonate.

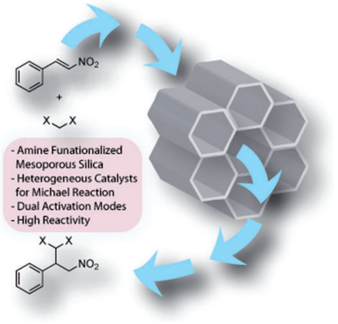

*ChemCatChem*
DOI: **10.1002/cctc.201200551**

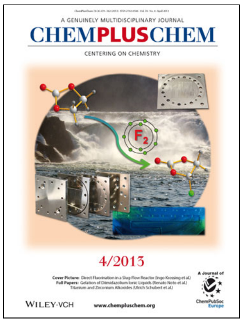

### Functionalized Nanoprobes

*A. R. Maity, A. Saha, A. Roy, N. R. Jana**

**Folic Acid Functionalized Nanoprobes for Fluorescence-, Dark-Field-, and Dual-Imaging-Based Selective Detection of Cancer Cells and Tissue**

**Probes with a preference**: Folate-functionalized quantum dots, gold/silver nanoparticles, and magnetic quantum dots have been synthesized and used as fluorescence, dark-field, and dual-imaging probes for the selective detection of cancer cells and tissues (see figure).

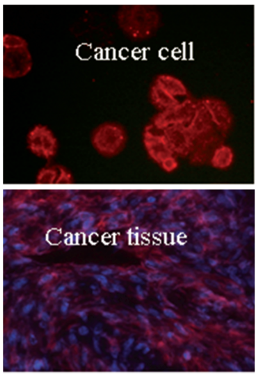

*ChemPlusChem*
DOI: **10.1002/cplu.201200296**

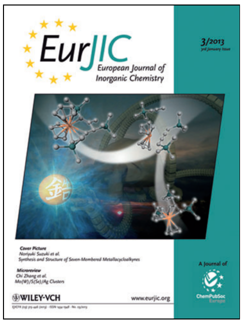

### Carborane Chemistry

*K. Z. Kabytaev, T. A. Everett, A. V. Safronov, Yu. V. Sevryugina, S. S. Jalisatgi, M. F. Hawthorne**

**B-Mercaptocarboranes: A New Synthetic Route**

A new synthetic route is described for the Pd-catalyzed preparation of mono- and di-B-mercapto(*ortho*-, *meta*-, and *para*-)carboranes from iodocarboranes and a sulfur nucleophile (TIPS–SH).

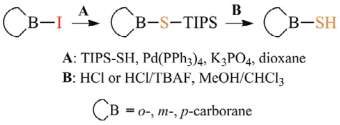

*Eur. J. Inorg. Chem.*
DOI: **10.1002/ejic.201201518**

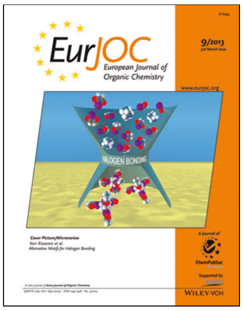

### (*E*)-Dehydroamino Acid Esters

*Y. Yasuno, M. Hamada, T. Yamada, T. Shinada,* Y. Ohfune**

**Stereoselective Synthesis of (*E*)-α,β-Dehydroamino Acid Esters**

A variety of (*E*)-dehydroamino acid esters were stereoselectively synthesized by using (diphenylphosphono)glycinate. The stereoselectivity was influenced by metal additives. Various (*E*)-dehydroamino acid esters were prepared by the condensation reaction of the new phosphonates with easily available aldehydes by choosing the appropriate reaction conditions.

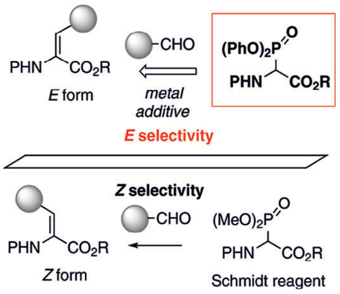

*Eur. J. Org. Chem.*
DOI: **10.1002/ejoc.201300112**

© 2013 Wiley-VCH Verlag GmbH & Co. KGaA, Weinheim

### Synthetic Methods

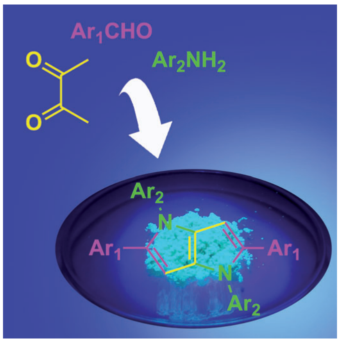

*A. Janiga, E. Glodkowska-Mrowka, T. Stoklosa, D. T. Gryko**

**Synthesis and Optical Properties of Tetraaryl-1,4-dihydropyrrolo-[3,2-*b*]pyrroles**

**Hit for six!** Six bonds are formed in a tandem process that gives rise to substituted 1,4-dihydropyrrole[3,2-*b*]pyrroles. Unparalleled simplicity and versatility of this one-pot reaction, no chromatography, as well as highly interesting optical properties have the potential to bring these molecules to the forefront in various applications.

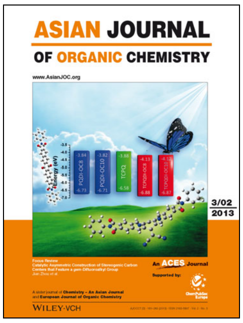

*Asian J. Org. Chem.*
DOI: **10.1002/ajoc.201200201**

### Food Chemistry

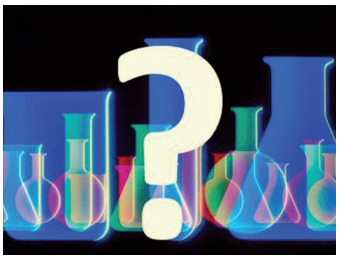

**Guess the Chemist (15)**

This person spent most of their career at the Max Planck Institute for Coal Research, Mülheim an der Ruhr, Germany, where they discovered a new principle for the separation of mixtures. This technique has found a lot of use in the food industry for the extraction of fats and oils from vegetable matter.Who are we looking for?

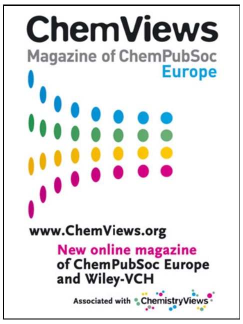

*ChemViews magazine*
DOI: **10.1002/chemv.201300029**

© 2013 Wiley-VCH Verlag GmbH & Co. KGaA, Weinheim
